# Decreased expression of circ_0020397 in intracranial aneurysms may be contributing to decreased vascular smooth muscle cell proliferation via increased expression of miR-138 and subsequent decreased KDR expression

**DOI:** 10.1080/19336918.2019.1619432

**Published:** 2019-05-28

**Authors:** Yushe Wang, Yong Wang, Yu Li, Bin Wang, Zhuang Miao, Xianzhi Liu, Yuanyuan Ma

**Affiliations:** a Department of Neurosurgery, Henan Provincial People’s Hospital, Zhengzhou, China; b Department of Neurosurgery, The First Affiliated Hospital, College of Medicine, Zhengzhou University, Zhengzhou, China; c Department of Anesthesiology, Henan Provincial People’s Hospital, Zhengzhou, China

**Keywords:** Intracranial aneurysm, circ_0020397, miR-138, KDR, cell proliferation

## Abstract

Dysfunction of vascular smooth muscle cells (VSMCs) mediates intracranial aneurysm (IA). KDR is reported to alleviate IA progression via promoting VSMC proliferation, while the upstream regulators are still unclear. Arterial wall tissues at the aneurysm site from 12 patients were obtained. The real-time PCR result indicated that circRNA_0020397 was down-regulated, but miR-138 was up-regulated in artery wall tissues and cells of IA. Overexpressed circRNA_0020397 promoted proliferation of human umbilical artery SMCs. MiR-138 negatively regulated KDR via binding with 3’UTR of KDR mRNA. The expression of circRNA_0020397 was negatively correlated with miR-138. In conclusion, our findings demonstrated that decreased expression of circRNA_0020397 in IA may contribute to the decreased VSMC proliferation via increasing miR-138 expression and subsequently decreasing KDR expression.

## Introduction

Intracranial aneurysm (IA) is a type of vascular disease that often leads to fatal vascular rupture and subarachnoid hemorrhage, which severely threatens human life. Even though numerous researches have been applied to the mechanism of IA, the morbidity and mortality are still increasing in recent years. Preliminarily, the pathogenic studies of IA are mainly focused on hemodynamic, arterial wall degeneration and genetic changes [1]. Recently, with the accumulation studies concerning immunization, non-coding RNAs, and epigenetics, the molecular mechanisms underlying the pathobiology of IA and the genetic risk factors have been widely identified [2,3]. For instance, Zhang *et al* demonstrated that overexpression of miR-448-3p prevented the development of IA through down-regulating macrophage-mediated inflammation [2]. Zhang *et al* indicated that the defects of regulatory T cells in IA were associated with the impairment in Tim-3 up-regulation [3]. However, the present studies still did not meet the needs of the clinic patients.

MicroRNA (miRNA), a class of non-coding RNA with the length of ~22 nucleotides, is the single stranded and short RNA is reported to mediate gene expression in post-transcriptional regulation and is widely presented in various cellular processes. It has been demonstrated that miRNAs are found to be involved in the progression of IA. For example, miR-29a is involved in regulating mitochondrial apoptotic pathway in IA [4]; miR-200a and miR-let-7b were significantly changed in plasma of IA patients [5]; miR-370 regulated human vascular smooth muscle cell proliferation in IA [6]. MiR-138 has been reported to regulate cell proliferation of human pulmonary arterial smooth muscle cells [7]. However, whether miR-138 is involved in regulating the proliferation of arterial smooth muscle cells in IA has been rarely reported. Notably, an online database Targetscan (http://www.rna-society.org) predicted that miR-138 could bind with the 3ʹUTR of KDR, which is a critical protein mediating cell proliferation of vascular smooth muscle cells and alleviating IA [6,8]. We supposed that miR-138 may affect the proliferation of arterial smooth muscle cells by the interaction with KDR.

Circular RNA (circRNA) is known as the covalently closed without containing a 5ʹ cap and 3ʹ polyA tail, and single-stranded transcripts of endogenous non-coding RNA is involved in many species [9,10]. CircRNAs present in eukaryotes, and stably composed by exons and introns. Additionally, the intracellular RNA regulatory networks of circRNA have been implicated its potential biomarkers in disease. Currently, the interaction between miRNAs and circRNAs has been demonstrated in various diseases. For example, hsa_circ_0020397 was first described in the colorectal cancer; the high expressed circ_0020397 promoted colorectal cancer cell growth via sponging miR-138 [11]. However, whether circ_0020397 affect the expression of miR-138 in IA is still unclear.

Herein, the present study aims to explore the interaction among circRNA_0020397, miR-138, and KDR on cell proliferation of vascular smooth muscle cells (VSMCs) in IA, implying the important role of circRNA_0020397 and miR-138 in VSMC proliferation in IA.

## Materials and methods

### Clinical samples

A total of 12 patients who were first diagnosed with IA using digital subtraction angiography (SDA) in the Henan Provincial People’s Hospital. The arterial wall tissues at the aneurysm site were isolated from IA patients during the surgery. The control samples of middle meningeal artery wall tissues were collected from 12 patients diagnosed with traumatic hematoma and tumors during the surgery. The present study was approved by the Ethics Committee of Henan Provincial People’s Hospital, and written informed consent was obtained from all participants.

### Isolation and culture of arterial smooth muscle cells

Arterial smooth muscle cells were isolated from arterial tissues of IA and non-IA patients by enzymatic hydrolysis as previously described [12]. Briefly, the arterial tissues were transferred into the Dulbecco’s Modified Eagle’s Medium (DMEM)/F12 medium dish, and carefully removed the connective tissues of the adventitia as much as possible by ophthalmology scissors. The blood vessels were cut longitudinally, and the inner wall was rinsed 1–2 times with DMEM/F12 medium followed by scraping the inner membrane removing the outer membrane. The smooth muscles were cut into small pieces of 1–2 mm^3^, and were placed in a sterile tube with ice-cold phosphate-buffered saline (PBS). Then the tube was fixed on Dynal MPC^TM^-1 (Invitrogen, Carlsbad, CA) magnetic particle concentration to allow the iron-containing placental tissue to be adsorbed to the inner wall of the tube, and the arterial tissues were resuspended with collagenase type 2 solution (80 U/L). The resuspended tissues were placed in a cell culture incubator for 100 mins, and the liquid was mixed every 30 mins to digest endothelial cells and extravascular tissues. The DMEM/F12 complete medium (containing 15% fetal bovine serum [FBS]) was added to passivate collagenase type 2, followed by two washes with warm PBS to wash away the digested endothelial cells and other cells, then resuspended in DMEM/F12 complete medium (containing 15% FBS) and incubated in a Petri dish designated plates 0. The phase difference microscope was used to observe the successful isolation of smooth muscle cells. The first generation is cultured for 12 to 15 days, and the second to sixth generation is 5 to 7 days.

### Real-time PCR

Total RNA was isolated using Trizol reagent, and the cDNA was synthesized using the HighCapacity cDNA Archive kit (Applied Biosystems, Life Technologies, Carlsbad, CA) in accordance with the manufacturer’s instructions. Real-time PCR was performed using SYBR Green qPCR Master Mix according to standard methods. The expression was calculated using the 2^−∆∆Ct^ method. The primers used in this study are listed as follows: hsa_circ_0020397 F: 5ʹ-GACCGTGAACCGAACCGTCATTTC-3ʹ; R: 5ʹ-TCATCCGCTCCTCTGGCATCATAG-3ʹ; miR-138 F: 5ʹ-ACACTCCAGC TGGGAGCTGGTGTTGTGAATCA-3ʹ; R: 5ʹ-CTCAACTGGTGTCGTGGAGTC GGCAATTCAGTTGAGCGGCCTG-3ʹ. The confirmation of circ_0020397 and miR-138 expression in human umbilical arterial smooth muscle cells was performed by using the primer sequences of circ_0020397 and miR-138.

### Western blot

Cells or tissues were lysed with loading lysis buffer. The protein was isolated after centrifugation. After quantifying the quality by BCA method, the protein was separated using 10% SDS-PAGE. Equal amount of protein was transferred onto PVDF and incubated with the primary antibodies (Abcam) at 4°C for 24 h. Then the membrane was incubated with the secondary antibody at room temperature for 1 h. The protein bands were visualized using the ECL kit (Beyotime, Shanghai, China).

### Cell culture

Human umbilical artery smooth muscle cells (HUASMCs) were purchased from Cell Systems (Kirkland, WA). Cells were cultured in DMEM and supplemented with 10% FBS. All cells were maintained at 37°C and 5% CO2, and the cultured HUASMCs were used at passage 6–7 in the following experiments.

### Cell transfection

Cell transfection was performed using Lipofectamine® LTX with Plus™ Reagent (Life Technologies). The indicated plasmids that used in this study were purchased from Sangon Biotech (Shanghai, China), and cells were transfected with the indicated plasmids according to the manufacturer’s protocol.

### Cell viability

Cell viability was performed using MTT assay in the present study. Cells were seeded in the 96-well plates, and incubated with 10 μL MTT (10 mg/mL) in the dark at 37°C for 2 h. The absorbance was detected at a wavelength of 570 nm.

### Cell apoptosis

Cell apoptosis was detected by the Annexin V Kit (Beyotime, Beijing, China). Cells were digested with 0.25% trypsin, and the digestion fluid was removed and cells were placed into the previously collected culture medium. After centrifuging at 12,000 RPM for 5 min at 4°C, the supernatant was discarded. Cell precipitation was collected to make cell suspensions using PBS. After another centrifugation and supernatant removal, 300 ml of Annexin VFITC and propidium iodide-labeled liquid were added to culture the cells at 4°C in the dark for 30 min. The flow cytometry (BD Pharmingen, San Diego, CA, USA) was used to detect cell apoptosis.

### BrdU experiments assay

Cell proliferation was determined using the BrdU incorporation assay. In brief, cells were seeded in a 6-well plate in DMEM containing 0.5% FBS for 24 h, and until cultured to 60–70% conﬂuences. BrdU Labeling and Detection Kit I (Hoffmann-La Roche, Basel, Switzerland) was used to BrdU incorporated into the cellular DNA by immunoﬂuorescence assay. Total cellular nuclei were stained with 4ʹ,6-diamidino-2-phenylindole (DAPI). The results were calculated as the percentage of BrdU-labelled cells.

### Luciferase reporter assay

The KDR 3ʹUTR promoter was amplified using real-time PCR, and then cloned into a pGL3-Basic plasmid (Promega, Madison, WI, USA). The constructed plasmid was transfected into cells using Lipofectamine 2000 (Life Technologies, Grand Island, NY, USA). After 36 h, cells were harvested and the luciferase activity was determined using the luciferase reporter assay system (TurnerBio Systems, Sunnyvale, CA, USA) according to the manufacturer’s instructions.

### Statistical analysis

All data in the present study were presented as means ± SD. Statistical analysis was analyzed using SPSS16.0 software (SPSS Inc., Chicago, IL, USA). The differences between groups were determined using *t*-test, while the differences between three or more groups were determined using ANOVA. P < 0.05 was depicted significant difference. All experiments were repeated at least three times.

## Results

### Circ_0020397 and KDR are down-regulated, but miR-138 is up-regulated in arterial wall tissues and arterial smooth muscle cells of IA

**Figure 1.**
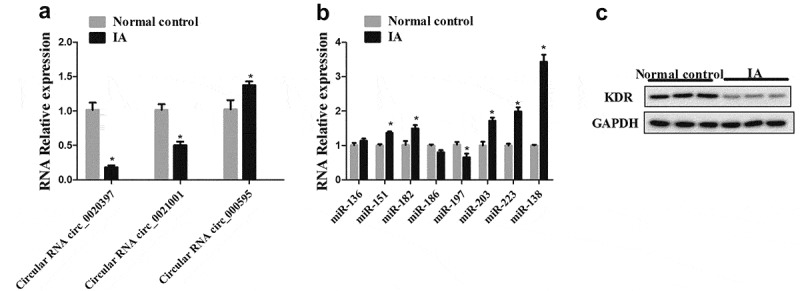
The expression of circRNA_0020397, miR-138 and KDR in clinical tissues. (a): Real-time PCR was performed to determine the expression of circRNA_0020397, circRNA_0021001, and circ_000595. (b): The expression of miR-136, miR-151, miR-182, miR-186, miR-197, miR-203, miR-223, and miR-138 was determined using real-time PCR. (c): The expression of KDR was measured using western blot. *p < 0.05 vs normal control.

In order to determine the different expressions in circ_0020397, miR-138, and KDR between IA patients and healthy control, the arterial wall tissues of IA and the healthy control were obtained. The expression of circ_0020397, circ_0021001 and circ_000595 in arterial wall tissues were all determined by real-time PCR. The result showed that the relative expression of circ_0020397 was markedly decreased in arterial wall tissues of IA in comparison with the control. (Figure 1(a)). Next, we examined the expression of miRNAs. The result revealed that miR-138 is significantly enhanced (Figure 1(b)). Additionally, the protein expression of KDR was significantly decreased in IA patients compared with the control (Figure 1(c)).

**Figure 2.**
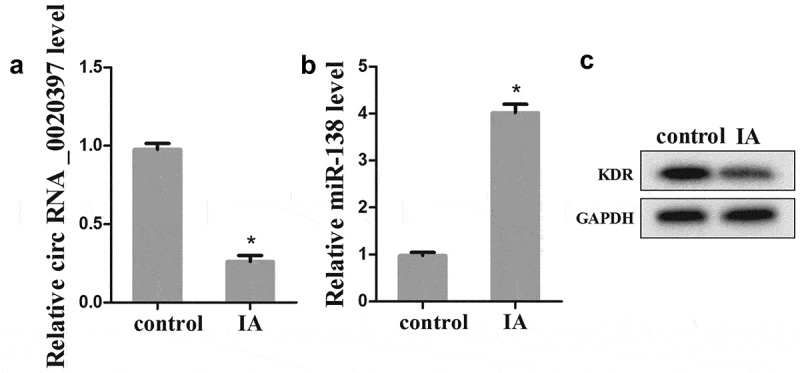
The expression of circRNA_0020397, miR-138 and KDR in isolated arterial smooth muscle cells. (a): The expression of circRNA_0020397 was determined using real-time PCR. (b): the expression of miR-138 was measured using real-time PCR. (c): the expression of KDR was detected using western blot. *p < 0.05 vs control.

To determine whether the expression of circ_0020397, miR-138, and KDR was changed in arterial smooth muscle cells of IA patients, the arterial smooth muscle cells were isolated from IA patients and the control. The results revealed that the expression of circ_0020397 was significantly decreased in comparing with the control (Figure 2(a)). The expression of miR-138 was markedly increased (Figure 2(b)), but the protein expression of KDR was decreased (Figure 2(c)).

### Overexpressed circ_0020397 promotes cell proliferation, but overexpressed miR-138 promotes cell apoptosis of HUASMCs

**Figure 3.**
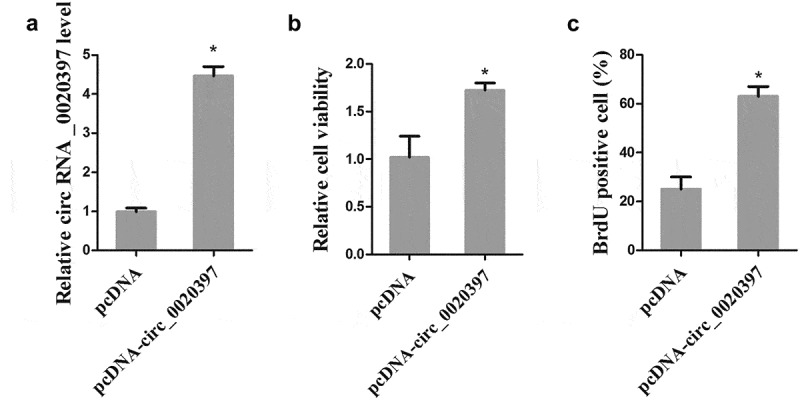
Effect of circRNA_0020397 on cell proliferation. HUASMCs were transfected with pcDNA or pcDNA-circRNA_0020397. (a): The expression of circRNA_0020397 was measured using real-time PCR. (b): Cell viability was determined using MTT assay. (c): Cell proliferation was determined by the BrdU experiment. *p < 0.05 vs pcDNA.

To examine whether circ_0020397 affects cell proliferation, the HUASMCs were transfected with pcDNA-circ_0020397 to overexpress circ_0020397. As presented in Figure 3(a), pcDNA-circ_0020397 transfection significantly promoted the expression of circ_0020397. Both cell viability and BrdU positive cells were significantly increased after circ_0020397 overexpression (Figure 3(b,c)). Meanwhile, we also transfected the HUASMCs with miR-138 mimic to overexpress miR-138, and cell apoptosis was determined by flow cytometry. The result demonstrated that HUASMC apoptosis was markedly increased by miR-138 overexpression (Supplemental Figure 1).

### Circ_0020397 is negatively correlated with miR-138 in HUASMCs

**Figure 4.**
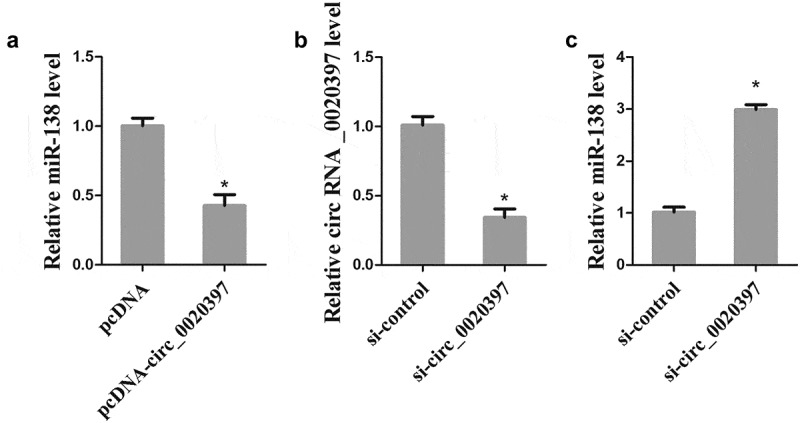
Role of circRNA_0020397 on regulating miR-138. (a): The HUASMCs were transfected with pcDNA-circRNA_0020397. Real-time PCR was performed to determine the expression of miR-138. (b) and (c): The HUASMCs were transfected with si-circRNA_0020397. Real-time PCR was performed to determine the expression of circRNA_0020397 and miR-138. *p < 0.05 vs pcDNA or si-control.

To elucidate the correlation between circ_0020397 and miR-138, HUASMCs were transfected with pcDNA-circ_0020397 or si-circ_0020397, and the expression of miR-138 was detected by real-time PCR. After the transfection with si-circ_0020397, the down-regulation of circ_0020397 in HUASMCs was confirmed (Figure 4(b)). The overexpressed circ_0020397 significantly suppressed miR-138 expression (Figure 4(a)), and the knockdown of circ_0020397 dramatically enhanced miR-138 expression (Figure 4(c)), suggesting that circ_0020397 expression is negatively correlated with miR-138expression in HUASMCs.

### MiR-138 regulates KDR expression by binding with the 3ʹUTR of KDR

**Figure 5.**
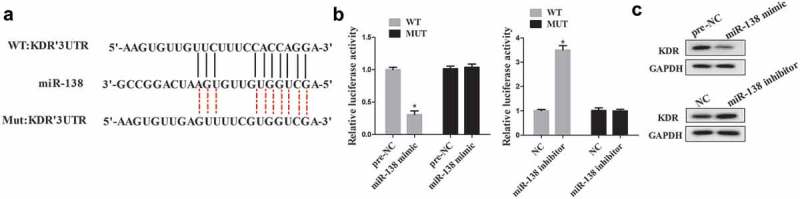
The interaction between miR-138 and KDR. (a): The predicted result of online TargetScan software. (b): Luciferase activity was determined using luciferase reporter assay. (c): Western blot was performed to determine the expression of KDR in cells transfected with miR-138 mimic/inhibitor. *p < 0.05 vs pre-NC or NC.

An online database Targetscan (http://www.rna-society.org) predicted that miR-138 could bind with the 3ʹUTR of KDR (Figure 5(a)). The luciferase reporter assay was performed to determine the relationship between miR-138 and KDR. The results showed that miR-138 mimic significantly suppressed luciferase activity in cells transfected with KDR 3ʹUTR-WT but not with KDR 3ʹUTR-MUT, while cells co-transfected with miR-138 inhibitor significantly promoted luciferase activity in cells transfected with KDR 3ʹUTR-WT but not with KDR 3ʹUTR-MUT (Figure 5(b)). Additionally, cells transfected with miR-138 mimic decreased the expression of KDR protein, while transfected with miR-138 inhibitor significantly promoted the expression of KDR protein (Figure 5(c)).

### Circ_0020397 contributes to regulate the expression of miR-138 and KDR

**Figure 6.**
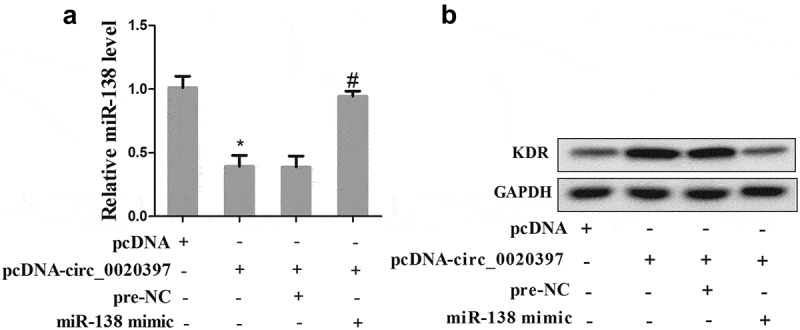
Role of circRNA_0020397 and miR-138/KDR. The HUASMCs were transfected with pcDNA-circ_0020397, or co-transfected with pcDNA-circ_0020397 and miR-138 mimic. (a): Real-time PCR was performed to determine the expression of miR-138. (b): Western blot was performed to determine the KDR level. *p < 0.05 vs pcDNA; #p < 0.05 vs pcDNA-circ_0020397+ pre-NC.

Then we further determined the role of circ_0020397 on affecting miR-138/KDR axis. HUASMCs were divided into 4 groups, including pcDNA, pcDNA-circ_0020397, pcDNA-circ_0020397+ pre-NC, and pcDNA-circ_0020397+ miR-138 mimic. The results revealed that overexpressed circ_0020397 suppressed the expression of miR-138 but promoted KDR. The co-transfection with pcDNA-circ_0020397+ miR-138 mimic restored the defect of miR-138 expression caused by circ_0020397 overexpression, while it reduced the enhancement of KDR protein caused by circ_0020397 overexpression (Figure 6(a,b)).

### Circ_0020397 promotes HUASMC proliferation partly through the miR-138/KDR axis

**Figure 7.**
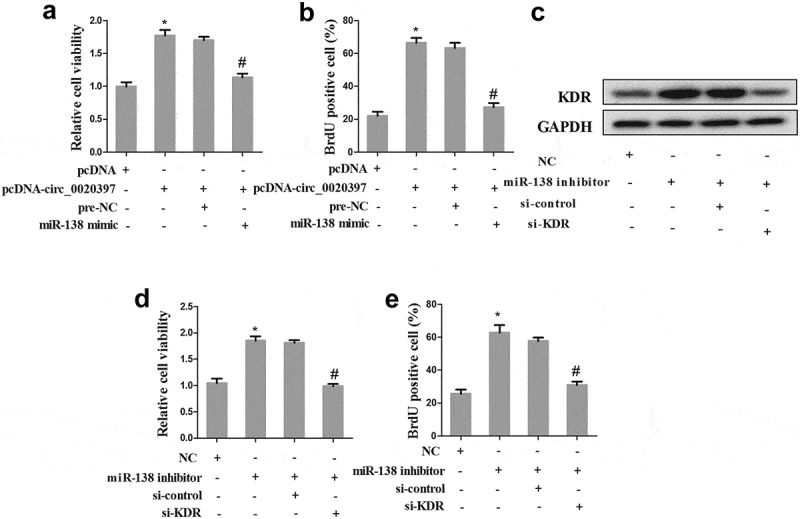
Role of circRNA_0020397/miR-138/KDR on cell proliferation. The HUASMCs were transfected with pcDNA-circ_0020397, or co-transfected with pcDNA-circ_0020397 and miR-138 mimic. (a): Cell viability was determined using MTT assay. (b): Cell proliferation was determined by the BrdU experiment. (c): The HUASMCs were transfected with miR-138 inhibitor, or co-transfected with miR-138 inhibitor and si-KDR. The expression of KDR was determined using western blot. (d): Cell viability was determined using MTT assay. (e): Cell proliferation was determined by the BrdU experiment. *p < 0.05 vs pcDNA or NC; #p < 0.05 vs pcDNA-circ_0020397+ pre-NC or miR-138 inhibitor +si-control.

The HUASMCs were divided into 4 groups as above. Cell viability and proliferation were determined in the 4 groups. The result revealed that the co-transfection with pcDNA-circ_0020397+ miR-138 mimic significantly decreased the enhancement of cell viability and proliferation raised by circ_0020397 overexpression (Figure 7(a,b)).

Additionally, the role of miR-138/KDR in cell proliferation was also determined. The HUASMCs were divided into 4 groups, including NC, miR-138 inhibitor, miR-138 inhibitor+si-control, and miR-138 inhibitor+si-KDR. Western blot revealed that miR-138 inhibitor promoted the expression of KDR, while cells co-transfected with miR-138 inhibitor and si-KDR reversed the effect of miR-138 inhibitor (Figure 7(c)). Additionally, the co-transfection with miR-138 inhibitor+si-KDR significantly decreased the enhancement of cell viability and proliferation raised by miR-138 inhibitor (Figure 7(d,e)).

## Discussion

IA can occur anywhere in the brain; most of them are located along a loop of arteries that run between the brain underside and the skull base. Vascular smooth muscle deletion or hypoplasia is found in a variety of cerebral aneurysms [13]. As an important channel on the cell surface, KDR contains a cerebral arterial current by electrophysiological experiments [14]. KDR, also known as VEGFR-2 is widely expressed in endothelial cells, and dominated the angiogenic response [15]. Furthermore, KDR plays an important role in neointima formation and adventitial angiogenesis [16,17]. The previous study has demonstrated that KDR mediated cell proliferation of vascular smooth muscle under hypoxia of venous-derived graft [8]. It is also a critical molecule in the proliferation of cerebral VSMCs, and the up-regulation of KDR promotes the cerebral VSMC proliferation [18]. Additional research identified that miR-370 targets KDR and regulating the activation of AKT signaling pathway in IA [6]. In the present study, we found that KDR was reduced in arterial wall tissues and isolated smooth muscle cells of IA, indicating the defect of KDR in the progression of IA.

Given that KDR promotes VSMC proliferation and alleviates IA, the upstream regulators of KDR are largely unknown. In recent years, the role of non-coding RNAs, including miRNAs and circRNAs, in the proliferation of VSMCs is gaining increasing attention. Notably, according to TargetScan prediction (http://www.rna-society.org), circ_0020397 and miR-138 may have a targeting relationship. Using the luciferase reporter assay, we confirmed that miR-138 negatively regulates KDR expression by binding with the 3ʹUTR of KDR mRNA. Furthermore, it has been reported that circ_0020397 regulates the downstream target of miR-138 by sponging miR-138. Therefore, the expression of circ_0020397, along with two IA-related circRNAs, circ_0021001 [19] and circ_000595 [20], was detected in arterial wall tissues of IA. The results demonstrated the significant expression change of the three circRNAs. Then we investigated the effect of circ_0020397 on VSMC proliferation, and found that the circ_0020397/miR-138/KDR pathway plays an important role in VSMC injury in IA.

MiR-138 has been widely studied in diverse diseases, such as non-small cell lung cancer [21], ovarian cancer [22], acute respiratory distress syndrome [23] as well as drug resistance in myeloma [24]. In addition to being a biomarker in those diseases, miR-138 is reported to regulate the progression of these diseases primarily by targeting the mRNA encoding key transcription factors. For example, miR-138 targets SP1 regulated cell proliferation in hepatocellular carcinoma [25]; SOX9 was targeted by miR-138 to regulated cell invasion in renal cell carcinoma [26]. In the present study, we found that miR-138 was significantly increased in arterial wall tissues and isolated smooth muscle cells of IA, and the luciferase reporter assay revealed that miR-138 targets KDR mRNA to regulate its expression.

Recently, studies on how competitive endogenous RNA (ceRNA) regulation of lncRNA-miRNA and circRNA–miRNA affected the occurrence and development of diseases has been receiving much attention. It has been reported that circRNA contains numerous response elements of miRNA and thus can exert a ceRNA function. Much study has reported the ceRNA of circRNAs, for example, circRNA-ACAP2 targets miR-21-5p to regulate the expression of Tiam1 and further modulated colon cancer progression [27]. The circRNA_0020123 sponged miR-144 to regulate the oncogenic properties of non-small cell lung cancer [28]. In the present study, circRNA_0020397 was significantly decreased in arterial wall tissues and isolated smooth muscle cells of IA. Additionally, the expression of circRNA_0020397 is negatively correlated with the expression of miR-138, implying the potential interaction between circRNA_0020397 and miR-138, although whether miR-138 is sponged by circRNA_0020397 in IA deserves further investigations.

Taken together, the present study implied the potential role of circRNA_0020397, miR-138, and KDR in the progression of IA. It showed that the decreased expression of circ_0020397 in IA may be contributing to decreased VSMC proliferation via increased expression of miR-138 and subsequent decreased KDR expression, which provides great help for further treatment exploration of IA.
