# Supplementary material for: Decreased expression of circ_0020397 in intracranial aneurysms may be contributing to decreased vascular smooth muscle cell proliferation via increased expression of miR-138 and subsequent decreased KDR expression
Source: Cell Adh Migr. 2019 May 28;13(1):220–8. doi: 10.1080/19336918.2019.1619432 (PMC6550538; doi:10.1080/19336918.2019.1619432)
Supplement: Supplemental Material [file kcam-13-01-1619432-s001.zip › Supplemental Figure 1 caption.docx]

**Supplemental Figure 1 Effect of overexpressed miR-138 on cell apoptosis.** The HUASMCs were transfected with miR-138 mimic, and cell apoptosis was determined by flow cytometry. *p<0.05 vs pre-NC.
